# Comparison of three boosting methods in parent-offspring trios for genotype imputation using simulation study

**DOI:** 10.1186/s40781-015-0081-1

**Published:** 2016-01-06

**Authors:** Abbas Mikhchi, Mahmood Honarvar, Nasser Emam Jomeh Kashan, Saeed Zerehdaran, Mehdi Aminafshar

**Affiliations:** Department of Animal Science, Science and Research Branch, Islamic Azad University, Tehran, Iran; Department of Animal Science, Shahr-e-Qods Branch, Islamic Azad University, Tehran, Iran; Department of Animal Science, Ferdowsi University of Mashhad, Mashhad, Iran

**Keywords:** Trios, Boosting methods, Imputation accuracy, Computation time

## Abstract

**Background:**

Genotype imputation is an important process of predicting unknown genotypes, which uses reference population with dense genotypes to predict missing genotypes for both human and animal genetic variations at a low cost. Machine learning methods specially boosting methods have been used in genetic studies to explore the underlying genetic profile of disease and build models capable of predicting missing values of a marker.

**Methods:**

In this study strategies and factors affecting the imputation accuracy of parent-offspring trios compared from lower-density SNP panels (5 K) to high density (10 K) SNP panel using three different Boosting methods namely TotalBoost (TB), LogitBoost (LB) and AdaBoost (AB). The methods employed using simulated data to impute the un-typed SNPs in parent-offspring trios. Four different datasets of G1 (100 trios with 5 k SNPs), G2 (100 trios with 10 k SNPs), G3 (500 trios with 5 k SNPs), and G4 (500 trio with 10 k SNPs) were simulated. In four datasets all parents were genotyped completely, and offspring genotyped with a lower density panel.

**Results:**

Comparison of the three methods for imputation showed that the LB outperformed AB and TB for imputation accuracy. The time of computation were different between methods. The AB was the fastest algorithm. The higher SNP densities resulted the increase of the accuracy of imputation. Larger trios (i.e. 500) was better for performance of LB and TB.

**Conclusions:**

The conclusion is that the three methods do well in terms of imputation accuracy also the dense chip is recommended for imputation of parent-offspring trios.

## Background

Innovations in genomic technologies provide new tools for enhancing productivity and wellbeing of domestic animals [1]. The technology can genotype some 10 million SNPs in an individual [2]. The availability of some thousands of SNPs spread across the genome of different livestock species opens up possibilities to include genome-wide marker information in prediction of total breeding values, to perform genomic selection [2]. Also a major challenge in implementing genomic selection in most species is the cost of genotyping [2]. Genotype imputation is an important process of predicting unknown genotypes, which uses reference population with dense genotypes to predict missing genotypes for both human and animal genetic variations at a low cost [2, 3]. Genotype imputation allows us to accurately evaluate the evidence for association at genetic markers that are not directly genotyped [4]. Analysis of un-typed SNPs can facilitate the localization of disease-causing variants and permit meta-analysis of association studies with different genotyping platforms [5]. As un-typed SNPs are not measured on any study subject, the missing information cannot be recovered from the study data alone [5]. To bring down genotyping costs, a reference population can be genotyped with a high-density panel while other animals are genotyped with a low-density panel in which markers are evenly spaced. Then, using information from the reference population, genotypes for un-typed loci can be inferred for individuals genotyped with the low-density panel [6]. Phasing and imputation methods can be divided into family-based methods (which use linkage information from close relatives) and population-based methods, which use population linkage disequilibrium information [6]. A “trio” data consist of genotypes from father-mother-child triplets and some phasing algorithms are adapted to be used in this type of data [7]. The accuracy of imputation depends on several factors, such as the number of SNPs in the low density panel, the relationship between the animals genotyped, the effective population size, and the method used [8]. Machine learning methods have been used in genetic studies to explore the underlying genetic profile of disease and build models capable of predicting missing values of a marker [9, 10]. Boosting is one of Machine learning methods for improving the predictive performance of classification or regression procedures which attempts to boost the accuracy of any given learning algorithm by applying it several times on slightly modified training data and then combining the results in a suitable manner [11]. Several methods of estimation have preceded boosting approach [12]. Common feature for all methods is that they work out by extracting samples of a set, calculating the estimate for each drawn sample group repeatedly and combining the calculated results into unique one. One of the ways, the simplest one, to manage estimation is to examine the statistics of selected available samples from the set and combine the results of calculation together by averaging them [11, 12]. The main variation between many Boosting Algorithms are the method of weighting training data points and hypotheses. Gradient boosting is typically used with decision trees of a fixed size as base learners [12]. In this research the accuracies of three different boosting methods i.e. (TotalBoost, LogitBoost, and AdaBoost) for imputation of un-typed-SNPs of parent-offspring trios are compared. The methods were compared in terms of imputation accuracy, computation time and factors affecting imputation accuracy. To evaluate the factors affecting imputation accuracy, sample size and SNP density were also examined.

## Methods

### The data simulation

**Fig. 1:**
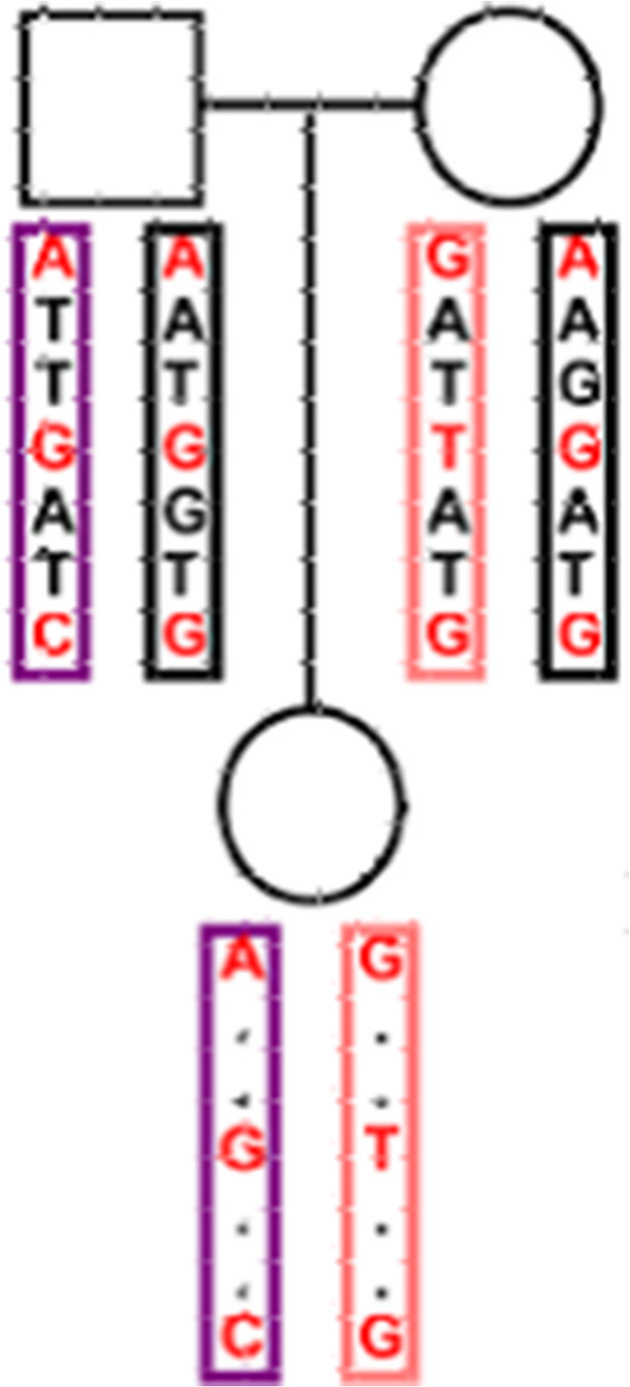
Genotype imputation within a trio

Four Data sets at different marker densities were simulated using the statistical software package R [13]. The R package hypred [14] was modified to simulate of data sets. A Historic Population (HP) was simulated that half of the animals were female and the other half male. Mating was performed during 50 generations using mutation rate of 2.5*10^−8^ per site by drawing the parents of an animal randomly from the animals of the previous generation. The considered genome comprised five chromosomes and each chromosome was set as 1 Morgan length. Different marker densities were created for each simulated data set. The number of SNPs per chromosome ranged from 1000 to 2000 in various datasets. The Reference population generated from the HP by mating parent groups. The parent groups were randomly selected from the last generation of the HP. Fifty percent of male offspring selected randomly from each group and were used as sires for the next generation. Also fifty percent of female offspring selected randomly as dams to produce the next generation and the mating scheme continued for 50 generations. The founder population randomly selected and the haplotypes of offspring generated them. Samples of 100 parent-offspring trios produced. Each sample was sequenced at depth of 5 k and 10 k. The sample size of the second set of simulations consisted 500 trios. Four different datasets of 100 trios with 5 k SNPs (G1), 100 trios with 10 k SNPs (G2), 500 trios with 5 k SNPs (G3), and 500 trio with 10 k SNPs (G4) were simulated. Bi-allelic SNPs were defined on each of homologous chromosomes and used “0” and “1” to denote the two alleles at each SNP site. The allele with high frequency was defined as ‘0’, and allele with low frequency as ‘1’ and an unknown value as ‘NaN’. Both parents genotyped for all SNPs, and offspring were genotyped for some of SNPs (low-density) (Fig. 1). For each of G1-G4 datasets five versions: NA10, NA30, NA50, NA70 and NA 90 were created with different levels of simulated missing data (10, 30, 50, 70 and 90 % of offspring genotypes). A total of 30 replicates of each simulated dataset were created.

## Imputation accuracy and running time

For each of the methods, the imputation accuracy per un-typed SNPs were calculated as the correlation between imputed and observed SNPs, then mean of imputation accuracy were calculated across the 5 replicates. Computation time were measured based on running each program in second on a windows server with 32 core CPU Intel, GPU: 192 CUDA Core and a total of 64 GB RAM by Profiler function in MATLAB.

## Assessment of factors affecting imputation accuracy

The SNP Density and sample size were considered as factors that could impact the imputation accuracy. For each dataset-imputation method combination, imputation accuracy were averaged across dataset versions NA10, NA30, NA50, NA70 and NA90 and referred as imputation accuracy. To assess the effect of the sample size on imputation accuracy, two groups of 100 and 500 parent-offspring trios were included the variation in SNP density. For both groups embedded simulated SNPs with two levels of 5 k and 10 K SNP panels and compared imputation accuracy based on trios sample size. The impact of each of these factors were assessed for each imputation method.

## Imputation methods

### SNP window

All the imputations in this study were done using MATLAB version (R2014a) [15]. The SNP window is defined by a fixed number of SNPs to the left and right (*L + R*) of the un-typed SNP (except when the un-typed SNP was near the end of a chromosome). A SNP window of size *L* corresponds to *L/*2 SNPs to the left and *L/*2 SNPs to the right of the un-typed SNP. In all imputation methods, a SNP window of size L centered at marker i to extend L markers left and right. For SNPs less than L markers from the beginning or end of a chromosome, the window extends L SNPs in one direction and to the boundary of the chromosome in the other. The distance defined in terms of the index of the SNP or the physical position on the chromosome, or the genetic distance. A distance measure fitted to the observed correlation matrix between markers and selected the best window size of 22 (for 1 k) and 10 (for 5 k and 10 k) for the imputation by scanning over a large range of windows. For all methods, the genotype datasets included a matrix *P* with m individuals and n SNP loci where the P *(i*, *j)* indicates the genotype of individuals at locus *i.* The target missing value is defined as *P* (*i, j*) = NaN. The individuals were assumed to have a known value at locus *i*, or otherwise it was excluded from the imputation but to be imputed in exactly the same way as sample *j*. On the other hand every other individuals had a known value at locus *i*, otherwise it was excluded from the imputation but to be imputed in exactly the same way as individuals *j.* In the imputation methods only parent genotype values at nearby SNP loci were used in the inference of *P (i*, *j*) in offspring.

## Boosting methods

### AdaBoost

The AdaBoost algorithm [16] is a well-known method to build ensembles of classifiers with very good performance [16]. It has been shown empirically that AdaBoost with decision trees has excellent performance, being considered the best off-the-shelf classification algorithm [16]. This algorithm takes training data and defines weak classifier functions for each sample of training data. Classifier function takes the sample as argument and produces value 0 or 1 in case of a binary classification task and a constant value - weight factor for each classifier. Generally, AdaBoost has shown good performance at classification. The sensitivity to noisy data and outliers is a weak feature of AdaBoost. Let X be a set of imputed SNPs, and y be a vector of observed (‘true’) SNP at an individual. Define M = 100 to be the number of independent classifiers (i.e. the imputation software). Given a training set of N SNP, there are Z = [(x_1_, y_1_), …,(x_i_, y_i_), …,(x_N_, y_N_)], where x*i* ∈ X = (x_i1_, x_i2_, x_i3_|i = 1,2, …, N), y*i* ∈ y = (a_1_, a_2_), and a_1_, a_2_ are the two alleles at a SNP locus, in question, for SNP i in the training sample.

**Initialize**: each SNP was assigned with an equal weight and

$$ {\mathrm{w}}_{\mathrm{i}}=1/N,\ i\ \in\ \left\{1, \dots,\ N\right\} $$

**Training**: For m = 1, 2… M classifiers

Call classifier m, which in turn generates hypothesis P_W_ (i.e. inferred SNPs in the training set). Calculate the error of P_W_:

Fit the class probability estimate

*P*_*m*_(*x*) = *P*_*w*_(*y* = 1|*x*) ∈ [0, 1], using weight w_i_ on the training data.

Set $$ {H}_m=0.5\  \log \left(\frac{1-{P}_m(x)}{P_m(x)}\right)\ \in\ R $$

Update the weight distribution W_i_ for next classifier as

Set *w*_*i*_ ← *w*_*i*_ exp(−*w*_*i*_*H*_*m*_(*x*_*i*_)) and renormalize to ∑_*i*_*w*_*i*_ = 1

**Testing**: In the testing set, each Un-typed SNP is classified via the so-called ‘weighted majority voting’. Briefly, the wrapper program is

$$ \mathrm{Output}\ \mathrm{H}\left(\mathrm{x}\right) = \mathrm{sign}\left({\displaystyle {\sum}_m^m{H}_m(x)}\right) $$

Above, the algorithm maintains a weighted distribution W_i_ of training samples x_i_, for i = 1, …,N, from which a sequence of training data subsets Z_m_ is chosen for each consecutive classifier (package) m. Initially, the distribution of weights is uniform, meaning that all samples contribute equally to the error rate. Next, the logit *H*_*m*_ of the rate of correctly classified samples is calculated for classifier m. A higher *H*_*m*_ is an indicator of better performance. For instance, when *H*_*m*_ = 0.5, *H*_*m*_ takes the value 0, and increases as *H*_*m*_ → 0 [16].

## LogitBoost

LogitBoost is a boosting algorithm that introduces a statistical interpretation to AdaBoost algorithm by using additive logistic regression model for determining classifier in each round [12]. Logistic regression is a way of describing the relationship between one or more factors, in this case instances from samples of training data, and an outcome, expressed as a probability. In case of two classes, outcome can take values 0 or 1. Probability of an outcome being 1 is expressed with logistic function. LogitBoost is a method to minimize the logistic loss, AdaBoost technique driven by probabilities optimization. This method requires care to avoid numerical problems [12].

### logitBoost algorithm for classification

1. Initialize the weights w_i_ = 1/*N*, *i* ∈ {1, …, *N*}
2. For m = 1 to M and while *H*_*m*_ ≠ 0
  - Compute the working response *z*_*i*_ = *y*_*i*_ − *P*(*x*_*i*_)/*P*(*x*_*i*_)(1 − *P*(*x*_*i*_)) and weights ${w}_i = P\left({x}_i\right)\left(1-P\left({x}_i\right)\right)$
  - Fit *H*_*m*_(*x*) by weighted least – squares of *z*_*i*_ to *y*_*i*_ with weights *w*_*i*_
  - Set H(x) = H(x) + 0.5 *H*_*m*_(*x*) and P(X) = $$ \frac{ \exp \left(H(x)\right)}{ \exp \left(H(x)\right)+ \exp \left(-H(x)\right)} $$
3. Output H(x) = sign (∑_*m*_^*m*^*H*_*m*_(*x*))

## TotalBoost

General idea of Boosting algorithms, maintaining the distribution over a given set of examples, has been optimized. A way to accomplish optimization for TotalBoost is to modify the way measuring the hypothesis goodness, (edge) is being constrained through iterations. AdaBoost constrains the edge with the respect to the last hypothesis to maximum zero. TotalBoost method is “totally corrective”, constraining the edges of all previous hypotheses to maximal value that is properly adapted. It is proven that, with adaptive edge maximal value, measurement of confidence in prediction for a hypothesis weighting increases [12].

The Boosting Algorithms in this study were AdaBoost, LogitBoost and TotalBoost which used the decision trees as learner [12, 17]. The main tuning parameter, the optimal number of iterations (or trees), determined and then the fitensemble function of MATLAB selected and set the number of decision trees to 100 for all boosting methods.

## Result and discussion

### Imputation accuracies

The imputation accuracies in different datasets are shown in Table 1 for ADA, LB and TOT. The accuracy of Imputation was high for all Boosting methods. For all data sets, imputation accuracies always decreased as the level of missing data increased. In general TOT had the lowest imputation accuracy compared to other Boosting methods. The results indicate that LB had the highest accuracy. A possible reason that TotalBoost was less accurate than other methods is that the datasets that used in the experiment may have violated multivariate normality. In addition, increasing the total number of trees can improve boosting ability to impute the un-typed SNP. Nevertheless other reason that affect the decrease of accuracy may be due to total number of trees that we used in the experiment. It was found that LogitBoost had higher accuracy than AdaBoost and TotalBoost algorithms because of LogitBoost was less sensitive to outliers and unlike AdaBoost, which uses an exponential function, LogitBoost uses the binomial log likelihood, which increases linearly rather than exponentially for strong negative margins. Because of this, LogitBoost is more robust than AdaBoost when data are noisy or samples are mislabelled [11]. However, LogitBoost can give better performance than AdaBoost and TotalBoost to impute the un-typed SNP. The imputation accuracy obtained of this research is not comparable with the other studies. Because in each study different population structure, levels of missing data and levels of LD between markers are assumed [18].

**Table 1:** Mean of imputation accuracy for Boosting methods in various versions on the four different datasets

Data set	Density	Sample size	Version	AB	LB	TB
	5 k	100	NA10	0.9843	0.9954	0.9611
	5 k	100	NA30	0.9883	0.9947	0.9638
G1	5 k	100	NA50	0.9822	0.9909	0.9621
	5 k	100	NA70	0.9777	0.9829	0.9583
	5 k	100	NA90	0.9211	0.9303	0.9246
			**Mean**	**0.9707**	**0.9788**	**0.9539**
	10 k	100	NA10	0.9861	0.9981	0.9702
	10 k	100	NA30	0.9886	0.9978	0.9697
G2	10 k	100	NA50	0.9912	0.9970	0.9679
	10 k	100	NA70	0.9898	0.9939	0.9647
	10 k	100	NA90	0.9653	0.9714	0.9523
			**Mean**	**0.9842**	**0.9916**	**0.9649**
	5 k	500	NA10	0.9859	0.9967	0.9650
	5 k	500	NA30	0.9885	0.9952	0.9650
G3	5 k	500	NA50	0.9877	0.9926	0.9638
	5 k	500	NA70	0.9800	0.9848	0.9618
	5 k	500	NA90	0.9288	0.9383	0.9362
			**Mean**	**0.9741**	**0.9815**	**0.9583**
	10 k	500	NA10	0.9787	0.9983	0.9706
	10 k	500	NA30	0.9799	0.9977	0.9692
G4	10 k	500	NA50	0.9830	0.9967	0.9665
	10 k	500	NA70	0.9877	0.9959	0.9634
	10 k	500	NA90	0.9706	0.9767	0.9552
			**Mean**	**0.9799**	**0.9930**	**0.9649**

NA10: 10 % of genotype is missing per offspring, NA30: 30 % of genotype is missing per offspring, NA50: 50 % of genotype is missing per offspring, NA70: 70 % of genotype is missing per offspring, NA90: 90 % of genotype is missing per offspring, Bold: Mean of different versions in each dataset

*AB* AdaBoost, *LB* LogitBoost, *TB* TotalBoost

### SNP density

**Fig. 2:**
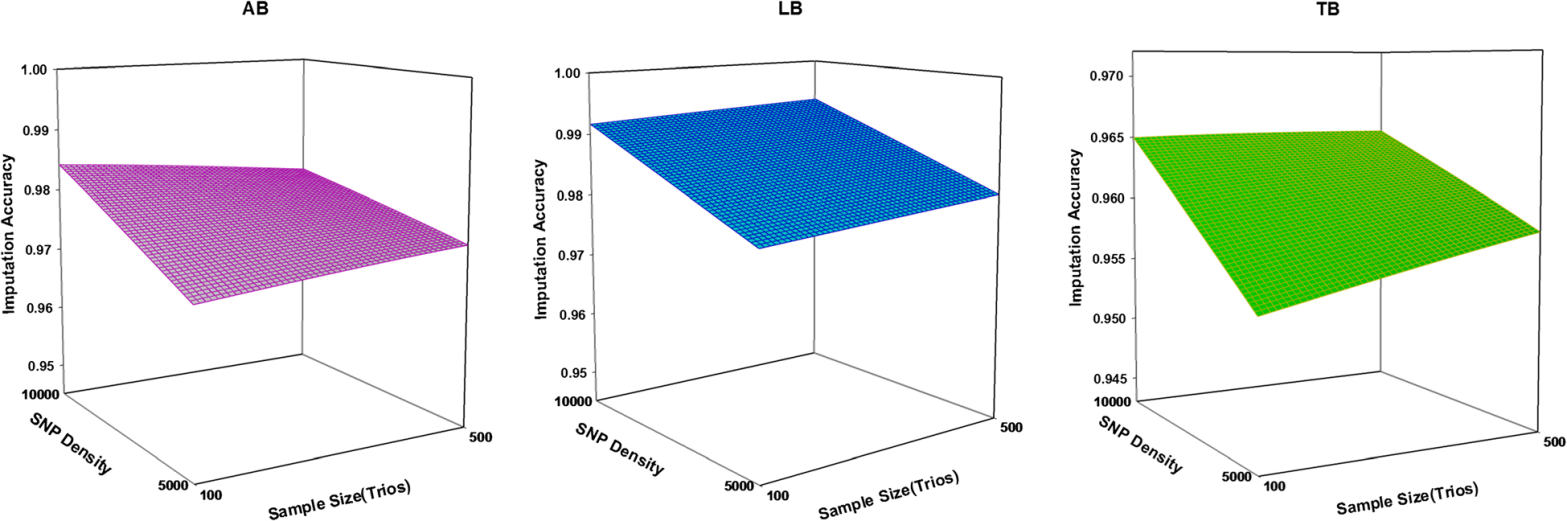
The effect of the sample size and SNP density on imputation accuracy

The accuracy of imputation increased with the number of SNPs for all Boosting methods examined. The imputation accuracy was lower for all levels of 5 K SNP panel compared to 10 k panels. Increasing the SNP density increased imputation accuracy for two sample size of trio (100 and 500), especially from 5 k to 10 k. There was a large increase in the imputation accuracy when using 10 k SNP panels. As a general trend, mean of imputation accuracy increased with increasing SNPs density and increasing sizes of trios (Fig. 2). It seems that imputation accuracy in all methods more influenced by the SNP density than sample size. Similar to the current results, Weigel et al. [19] reported mean imputation accuracy from 80 to 95 % when animals were genotyped with a medium-density panel (2000–4000 SNPs); less than 80 % when animals were genotyped for 1000 SNPs or less, and greater than 95 % when animals were genotyped for more than 8000 SNPs. All Boosting methods had better performance on the high density dataset (10 k). We believe this is reasonable since a higher density provides more neighboring SNPs, and consequently greater linkage disequilibrium, for imputation purpose [20].

### Sample size

The accuracy of imputation increases for all methods under the condition of low SNP density (5 k), as the number of trios increase. The results show that under the condition of high SNP density (10 k), accuracy of imputation increased for LB and TB as the number of trios increased. The imputation accuracy for AdaBoost (AB) in 10 K SNP panel was slightly lower. It seems that AdaBoost is suitable for imputation of un-typed SNP in small sample size. However, the effect of the sample size on imputation accuracy is less than effect of SNP density on imputation accuracy. The results show that the sample size of the trios is a substantial impact on imputation accuracy. We have demonstrated with G3 and G4 datasets that the use of 500 trios produced substantial gain in imputation accuracy and improved imputation accuracy for LB and TB. The larger sample size will produce more consistent estimates of measured parameters, resulting in improved imputation accuracy for various methods [21]. The performance of any classification depends on sample size, which may be especially so for present methods, since the number of parameters to be estimated is large and low sample size may lead to unstable results [22]. It was found that larger trios (i.e. 500) could help to better performance of LB and TB and could be suitable for imputation of un-typed SNPs [23]. The LB and TB showed the large changes with increasing the number of trios. It is concluded that these methods are suitable for imputation of un-typed SNP in large sample.

### Computation of time

The detailed runtime of the all three methods on four datasets at missing rate of 90 % (NA90) presented in Table 2. For all data sets, the AB was the fastest algorithm and LB was next fastest. The TB was always the slowest and needed more time to impute a dataset. An important factor in evaluating machine learning algorithms is how quickly their runtime increases with sample size of dataset. As number of trios grow, the speed of all eight methods needed some more time to impute a dataset, especially for large SNP panel. AdaBoost required less computer time than the other boosting methods, which may be an advantage among boosting methods when using large data sets with several thousand markers. The TotalBoost algorithm seemed to be too time-consuming in large data sets and it has lowest imputation accuracy than other methods. The computing time changed with increasing the sample sizes. Increase of sample size from 100 to 500 resulted, the computing time of all methods increased.

**Table 2:** Average imputation runtime on four datasets (seconds)

Data set	Sample size	Density	Version	AB	LB	TB
G1	100	5 K	NA90	2930	3055	6975
G2	100	10 K	NA90	6511	6788	13956
G3	500	5 K	NA90	3460	3665	10221
G4	500	10 K	NA90	7601	7802	23521

NA90: 90 % of genotype is missing per offspring

*AB* AdaBoost, *LB* LogitBoost, *TB* TotalBoost

## Conclusion

In this study we compared the performance of three Boosting methods based imputation of parent-offspring trios in terms of imputation accuracy, computation time and factors affecting imputation accuracy. Simulation of datasets showed the methods performed well for imputation of un-typed SNPs. The LB had the highest accuracy of the three imputation methods examined. Accuracy of imputation increased with the increase of the number of SNPs and trios. The 10 K SNP panels can be imputed with high accuracies than 5 k SNP panels. In terms of imputation time, AB outperformed LB and TB. The LB and TB methods are suitable for imputation of un-typed SNP in large samples. The results indicated that the methods are suitable in terms of imputation accuracy and denser chip is recommended for imputation of parent-offspring trios.
